# Haplo2Ped: a tool using haplotypes as markers for linkage analysis

**DOI:** 10.1186/1471-2105-12-350

**Published:** 2011-08-22

**Authors:** Feng Cheng, Xianglong Zhang, Yinan Zhang, Chaohua Li, Changqing Zeng

**Affiliations:** 1 Laboratory of Disease Genomics and Individualized Medicine, Beijing Institute of Genomics, Chinese Academy of Sciences, No.7 Beitucheng West Road, Beijing 100029, PR China; 2 Institute of Vegetables and Flowers, Chinese Academy of Agricultural Sciences, No.12 Zhongguancun South Street, Beijing 100081, PR China; 3 Graduate School of the Chinese Academy of Sciences, No.19A Yuquan Road, Beijing 100049, PR China

## Abstract

**Background:**

Generally, SNPs are abundant in the genome; however, they display low power in linkage analysis because of their limited heterozygosity. Haplotype markers, on the other hand, which are composed of many SNPs, greatly increase heterozygosity and have superiority in linkage statistics.

**Results:**

Here we developed Haplo2Ped to automatically transform SNP data into haplotype markers and then to compute the logarithm (base 10) of odds (LOD) scores of regional haplotypes that are homozygous within the disease co-segregation haploid group. The results are reported as a hypertext file and a 3D figure to help users to obtain the candidate linkage regions. The hypertext file contains parameters of the disease linked regions, candidate genes, and their links to public databases. The 3D figure clearly displays the linkage signals in each chromosome. We tested Haplo2Ped in a simulated SNP dataset and also applied it to data from a real study. It successfully and accurately located the causative genomic regions. Comparison of Haplo2Ped with other existing software for linkage analysis further indicated the high effectiveness of this software.

**Conclusions:**

Haplo2Ped uses haplotype fragments as mapping markers in whole genome linkage analysis. The advantages of Haplo2Ped over other existing software include straightforward output files, increased accuracy and superior ability to deal with pedigrees showing incomplete penetrance. Haplo2Ped is freely available at: http://bighapmap.big.ac.cn/software.html.

## Background

Linkage analysis plays an important role in mapping disease-causing genes. Compared to other methods, such as association research, not only are very limited samples needed in linkage study, but also the high disease homogeneity among pedigree members increases the possibility of locating causative genes [1,2]. Furthermore, linkage mapping of complex traits was made feasible for experimental organisms, such as animals and plants, through the use of genetic mapping in large crosses [3,4]. Linkage analysis has wide applications in both medical experiments and agricultural breeding.

Along with the achievement of high-throughput SNP genotyping, using whole genome SNP data for linkage analysis has been shown to be an efficient strategy [5,6]. However, because of their two-allele character, the heterozygosity of SNP markers is usually lower than traditional genetic markers, such as short tandem repeats (STRs). Therefore, two point linkage analysis with SNP data is often insufficiently powerful. Considering the abundance of SNPs in the human genome, the use of multi-point based methods, such as haplotype-disease co-transmission analysis, would largely overcome the low heterozygosity of individual SNPs, because haplotypes formed by multi SNPs could easily achieve the maximum heterozygosity in pedigrees.

Software packages have been developed to carry out multi-point analysis. The traditional linkage methods employed two basic algorithms: the Elston-Steward algorithm, used in Allegro, and the Lander-Green algorithm, used in Merlin. SNPLINK, a Perl Script that performs full genome linkage analysis, uses both these algorithms. However, the application of these two algorithms is limited, either by the number of markers or by the size of the pedigrees. Another program, SNP4Linkage, is based on allele sharing determination and is better adapted to high-density SNP genotyping data. Nevertheless, it still lacks a tool for considering haplotype fragments as genomic markers for linkage research [7-10]. Therefore, Haplo2Ped was developed. It can perform whole genome linkage analysis with haplotypes and generate a corresponding report file that contains linkage regions, LOD scores, and the candidate genes. To help users to obtain further information, links for the candidate genes to databases of gene annotations and OMIM (Online Mendelian Inheritance in Man) are also offered [11]. Meanwhile, an auto-generated 3D picture allows users to visualize the linking signals clearly on a genomic scale.

## Implementation

**Figure 1:**
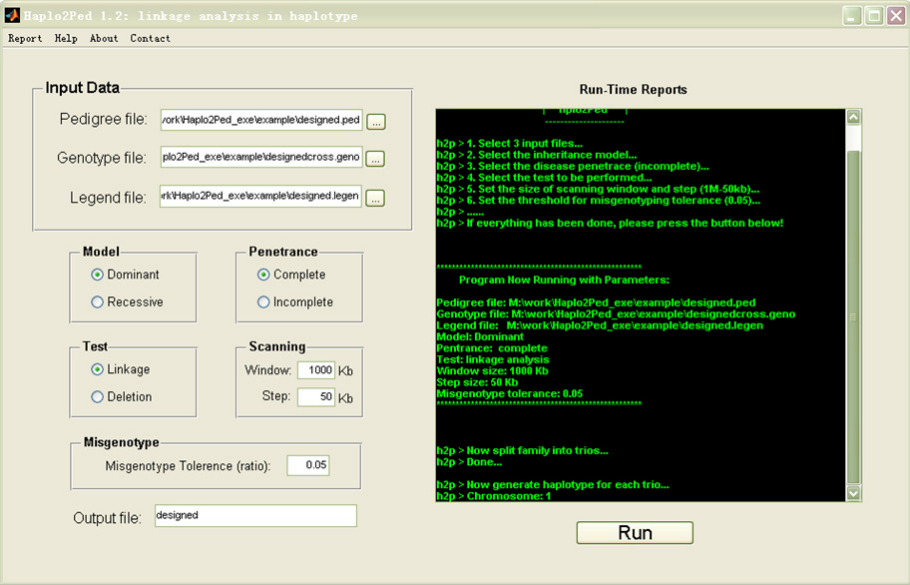
**The user-friendly interface of Haplo2Ped**. Users select input files and set parameters on the left side. The Run-Time Reports on the right side indicate the ongoing step of the process.

**Figure 2:**
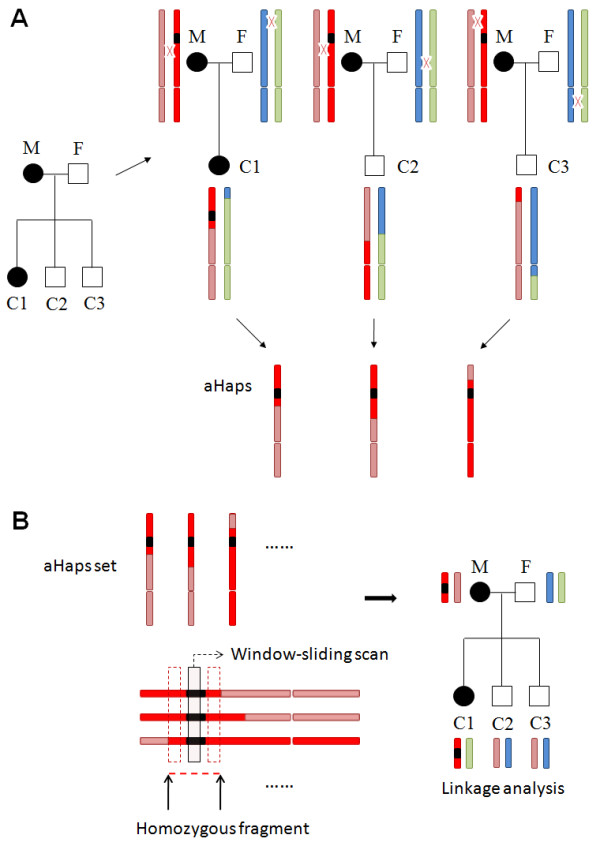
**The nuclear trios division and haplotype sharing analysis**. (**A**) The family is divided into three nuclear trios (father, F; mother, M; child, C). Parental haplotypes are then deduced from the genotype data of these trios. The one co-segregating with the disease is named as an aHap (affected haplotype) and submitted into homozygosity analysis. (**B**) A windows slides along the whole genome to determine homozygous fragments. LOD scores are calculated using homozygous aHap regions as markers. Bars represent chromosomes; the black region is the causal mutation of the disease; red crossovers between parental chromosomes denote recombination events; the designed affected status is shown by filled circles/boxes for affected and open circles/boxes for unaffected.

Haplo2Ped is an effective tool using haplotypes as markers for linkage analysis. It is well-suited for genome-wide linkage mapping with high density SNP data. It provides a user-friendly interface to select input files and set parameters to perform the analysis (Figure 1). The Run-Time Reports illustrate the processing phases of the entire analysis. For the algorithm, it divides the studied family into several small nuclear trios (father, mother, and one child) (Figure 2A). The parental haplotypes of a haploid gamete can then be deduced from the genotype data of these trios, according to Mendelian inheritance rules. During this process, SNPs showing Mendelian errors are automatically removed. Genotypes that are heterozygous in all three members of a trio are treated as uninformative data, because it is difficult to phase the haplotype at these sites in this trio. Based on the affected status and the inheritance model, the parental haplotypes that co-segregate with the disease are selected and named as aHaps (affected haploids) (Figure 2A). These aHaps are then submitted for haplotype sharing analysis.

Firstly, we consider the example of a dominant disease model. In a trio, if the child and his father are both affected, the father's transmitted haploid will be selected as an aHap. Conversely, if the child is healthy, the affected father's untransmitted haploid will be deemed as an aHap. When we cannot be sure of the child's affected status (the child is too young to show symptoms or it is a disease with incomplete penetrance), then the affected father's two chromatids would be selected and treated with the rule that at least one of them is an aHap.

Once the set of aHaps is determined from all the trios, the haplotype sharing analysis is performed. A window-length and step-size are set to scan these aHaps to determine disease candidate segment(s) generated from recombination events (Figure 2B). For the haplotypes locating within the same scanning window, if they show homozygosity in all aHaps, this window would be merged with the adjacent homozygous windows until the sliding window process moves out of the area showing homozygosity. After the completion of aHap scanning, the family's haplotype fragments that are located in the homozygous aHap regions are determined and are consequently used as markers to calculate LOD scores [12].

For a disease with incomplete penetrance, we cannot determine whether the asymptomatic healthy child is really disease free or not. As referred to above, we treat both the transmitted and un-transmitted haploids of their affected parent as paired aHaps. The two assumed aHaps are then compared to the assured aHaps. A true disease co-segregation haplotype fragment should be found in at least one of the two assumed aHaps. Regarding determination of a candidate region by window sliding, although paired aHaps are not as informative as the assured aHaps, they may still contribute to shortening the linked regions and identifying whether or not the child carries the disease targeted haplotype.

Using the above method to analyze a disease caused by fragment deletion may result in two linked regions separated by a homozygous region caused by the deletion. For large deletions (> 500 Kb), such a result may lead to confusion or an incorrect conclusion. Therefore, Haplo2Ped provides a LOH (loss of heterozygous) test to detect large fragment deletions.

## Results

**Figure 3:**
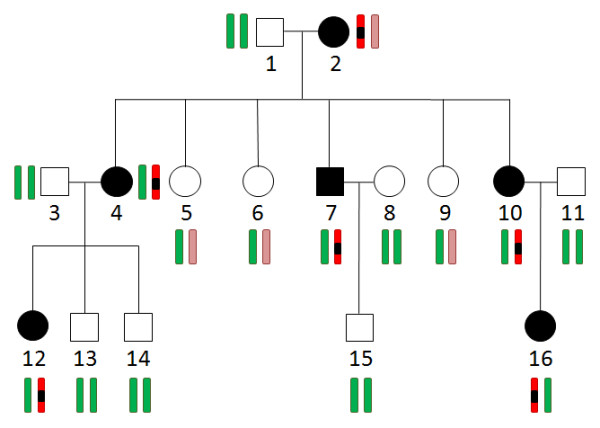
**The pedigree structure of the simulated example**. Squares (males) and circles (females) filled with black indicate affected members. Individual 2 is the disease founder. The disease is assumed to fit an autosomal dominant model with complete penetrance. Bars denote chromosome groups of each member; red bars are groups that were simulated to take the disease-causal mutations (black regions); other colored bars, including green and light brown, are the unaffected chromosome groups.

We used the pedigree data generated from Illumina 370 K CNV-Quad chip as an example for analysis. The raw dataset came from a family with RP (retinitis pigmentosa) disease. To test Haplo2Ped, we made certain changes in the raw data to generate simulated disease targets. The pedigree structure is shown in Figure 3. Six independent chromosome regions were assumed to carry the mutations (Expected Regions in Table 1). They were initialized with lengths of 1, 3, 4, 5, 8, and 10 Mb in chromosome 1, 5, 9, 13, 17, and 21, respectively. For the two regions in chromosome 17 and 21, crossovers were deliberately designed in the middle of them. Therefore, the final sizes of the disease carrying haplotypes were 1, 3, 4, 5, 4, and 5 Mb (see additional file 1: The pedigree simulation process).

**Table 1:** Simulated linkage regions and the regions detected by Haplo2Ped

		Haplo2Ped		Merlin		
			
Chr	Expected region^a^(bp)	Detected region (bp)	LOD score	Detected region (bp)	LOD score	SNPLINK^b^
1	216,655,820-217,662,693	216,631,505-217,703,573	3.010	216,907,787-217,662,692	1.773	217,005,036-217,668,715

5	38,764,018-41,787,459	38,631,518-42,305,421	3.010	38,854,039-41,712,049	1.777	38,886,647-42,163,690

9	27,316,060-31,341,194	27,143,875-31,616,167	3.010	27,319,623-31,394,530	1.777	27,306,972-31,369,980

13	96,346,535-101,352,382	96,316,072 101,404,566	3.010	96,328,971-101,304,534	1.778	96,836,579-101,356,932

17	50,661,600-54,645,983	50,621,012-54,818,763	3.010	50,690,828-58,850,202	1.804	50,661,601-54,726,213

21	21,812,513-26,812,202	21,781,794-27,090,473	3.010	21,833,903-26,812,201	1.778	21,812,514-26,818,797

1	/	/	/	55,489,086-62,400,262	1.778	/

5	/	/	/	10,529,760- 11,796,160	1.778	/

9	/	/	/	73,498,210- 80,047,165	1.778	/

^a^Expected region, the simulated region assumed to contain disease mutations.

^b^Regions detected by SNP4Linkage were the same as those reported by SNPLINK.

**Figure 4:**
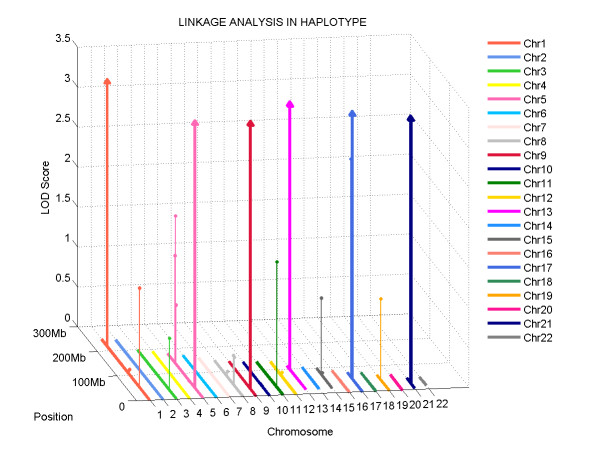
**Graphical report of the Haplo2Ped application**. The x- and y-axes represent the individual autosomes and their physical positions, respectively. The z-axis shows the LOD scores of each linkage region. The regions with the maximum LOD scores are labeled with arrowheads and others are labeled with circles at the top. In this example, all assumed regions were detected by Haplo2Ped with the highest LOD scores in chromosomes 1, 5, 9, 13, 17, and 21.

The genotype data of the simulated pedigree was then submitted to Haplo2Ped for linkage mapping. Figure 4 shows the linkage signals across the whole genome detected by this tool. All the six assumed regions were identified with a LOD score of 3.010, the maximum value at the genome-wide level. No other regions showed such a large LOD score, indicating that no false positives were generated by Haplo2Ped in this simulated analysis (see additional file 1: The pedigree simulation process). As shown in Table 1, these expected candidates are completely included in the regions found by Haplo2Ped, and each of the detected candidate regions is only slightly larger than the simulated region, suggesting that Haplo2Ped is reliable, sensitive, and accurate.

To compare the efficiency of Haplo2Ped with other existing software, we submitted the same simulated data to Merlin, SNPLink, and SNP4Linkage. The output results are listed in Table 1. Merlin reported the six regions co-segregating with the disease with a LOD score of around 1.78, which was the maximum value across the whole genome. Four of these six reported regions were smaller than the expected regions indicating that some regions that might harbor the disease-causal mutation were missed. Such a low LOD score could not reflect the real level of linkage between the disease-causal regions and the disease phenotype. Except for the six simulated regions, Merlin also detected three other regions with LOD scores of around 1.78 (Table 1). These false positive results could add to the difficulty in locating the disease-causing mutations in real studies. Moreover, Merlin reported the LOD score of every individual SNP. The LOD scores of SNPs on the border of the linked regions usually increase from a low value of unlinked regions to a high value of linked regions or decrease the other way around. Thus, another concern is that it is usually difficult for users to determine the borders of the regions detected by Merlin.

SNPLINK did not report LOD scores in the final output files although it showed good accuracy on four regions co-segregating with the disease. Furthermore, SNPLINK missed some regions on the left edge of two expected regions on chromosomes 1 and 13. The results from SNP4Linkage were the same as SNPLINK. There were no false positive regions detected by these two programs.

In a real study of a digital-anomaly family, we applied Haplo2Ped to SNP genotype data from 13 family members for the linkage analysis by haplotype, and successfully located the linkage region. Further study determined the mutation of the causative gene [13]. Comparisons of Haplod2Ped and other existing software using the data from the real study are listed in additional file 2: Software comparisons using real data. All the software packages successfully located the disease-causing region, while Merlin reported more false positive regions.

To evaluate the false positive rate of Haplo2Ped, we simulated genotype data sets for thirty pedigrees with one causal mutation each using an in-house developed Perl script (packaged with Haplo2Ped). Each data set was analyzed by both Haplo2Ped and Merlin. The false positive regions reported by Merlin were significantly more than those reported by Haplo2Ped (Figure S1 in additional file 3: Evaluation of false positive rate of Haplo2Ped with completely simulated genotype data), indicating that using haplotypes that are of high heterozygosity as markers has better efficiency in filtering false positive regions than using only individual SNPs.

## Discussion

The haplotype-sharing scanning of aHaps is the most important step in Haplo2Ped. For dominant diseases, the main point is to confirm whether the disease haploid is transmitted or not. In the case of recessive diseases, two haplotypes of the affected individual are both aHaps. Additionally, for either a dominant or recessive model, Haplo2Ped is only suitable for one-disease-founder cases (i.e. a disease with complete homogeneity). Two or more disease founders would result in more than one type of disease haplotype for the family, which could lead to either loss of linkage signals, or generate false positives. Haplo2Ped analysis is based on deduced parental haplotypes; therefore, in cases where one parent is missing in a nuclear family, it is still applicable for linkage study.

Our simulation analysis showed that Haplo2Ped was consistently accurate in pinpointing the regions co-segregating with the disease. It did not miss any expected regions, while other software reported biased results, especially on the left edge of certain regions. Given the limited recombination events accumulated in a pedigree, both the disease-causing mutation and the neighboring SNPs in a shared haplotype co-segregate with the disease phenotype. However, when a disease-causing haplotype is transmitted to offspring, recombination occurs at random sites of this haplotype, indicating that the disease-causing mutation also probably locates at the edge of our assumed regions. If any expected regions are missed, the risk of not locating the final mutation is increased.

A gain of LOD score using haplotypes as markers in our tool demonstrated an advantage over Merlin, which is based on classical maximum-likelihood methods. Employing haplotypes with high heterozygosity as markers avoided the false positive results generated by Merlin, which is subject to the low heterozygosity of individual SNPs. Furthermore, the LOD score of the SNPs reported by Merlin in the assumed regions usually varies over a wide range. Many SNPs even show lower LOD scores than those in the unlinked regions. This adds to the difficulty of locating the linked regions. Thus, we suggest that our method of combining the heterozygosity of multi-SNPs and the breakpoints of recombination (borders of co-segregating haplotype) better reflects the stable strength of a linkage region compared to a method that only uses the heterozygosity of individual SNPs.

Another advantage of Haplo2Ped is its capability of dealing with the diseases that exhibit incomplete penetrance, a model of which is not included in software such as SNPLINK. Using simulated data with incomplete penetrance, although Merlin reported expected linkage regions similar to those of Haplo2Ped (additional file 4: Software comparison with simulation data with incomplete penetrance), it generated three false positive regions, while Haplo2Ped reported none. Performance on the data from the real study with incomplete penetrance and the simulated genotype data of thirty pedigrees also showed that Merlin reported more false positive regions than Haplo2Ped. In addition, using the notion of shared affected haploids among affected individuals instead of traditional algorithms, such as the Elston-Steward and the Lander-Green algorithms means that Haplo2Ped is not restricted by the number of markers or the family members. The successful application of Haplo2Ped to a real study demonstrated its power in detecting the regions harboring the disease-causing mutation.

The haplotype-sharing analysis is sensitive to mis-genotyped SNPs, which may generate false breakpoints in the haplotype fragments. To prevent such errors, we use a window sliding method to scan the genome. For the haplotype fragment in each window, we determine if it is homogeneous among all aHaps with a certain tolerance. For example, we set the level of inconsistent SNPs to less than 5% of the total in the above analysis. When the window steps into the linkage region, the ratio of inconsistent SNPs should largely decrease and when the window steps into the recombination free region, the ratio quickly increases to above 5%. As the real ratio of mis-genotyped SNPs is usually unknown or is different in different genomic regions, we suggest a threshold of 5% be set as the mis-genotype tolerance. Generally, a 5% typing error is much higher than the true ratio in experiments, and it would generate a candidate region slightly larger than the real linkage region as seen in our example. Despite a relatively conservative setting, the introduction of false breakpoints by mis-genotyped SNPs should be prevented.

## Conclusions

The new software, named Haplo2Ped, which uses haplotype fragments as mapping markers in whole genome linkage analysis, has been developed. Comparison with other programs by simulation tests and successful application in a real study demonstrated its high efficiency and reliability. Haplo2Ped is not restricted by the number of markers or family members. Moreover, it also provides LOH (loss of heterozygosity) detection for pedigrees in which fragment deletion causes the disease. We propose that haplotype fragments could be powerful genomic markers in linkage analysis.

## Availability and requirements

Software name: Haplo2Ped

Software home page: http://bighapmap.big.ac.cn/software.html

Operating system(s): Windows or Linux

Programming language: Matlab platform

Other requirements: No

License: No

## Competing interests

The authors declare that they have no competing interests.

## Authors' contributions

FC and CZ conceived the study. FC and XZ wrote the paper. FC implemented the program. XZ carried out comparison analysis between different software. YZ and CZ improved the manuscript. CL conducted the microarray experiments. All authors read and approved the final manuscript.

## Supplementary Material

Additional file 1 **The pedigree simulation process**. The pedigree simulation process was carried out based on a real genotype data set of a RP (retinitis pigmentosa) family. The family members' affected status was reset and six disease co-segregating haplotypes were simulated in chromosomes 1, 5, 9, 13, 17, and 21. Click here for file

Additional file 2 **Software comparisons using real data**. Linkage regions detected by three different softwares are from data of a real study (Table S1). The pedigree analyzed is a three-generation Han Chinese family with complex digital anomalies. Click here for file

Additional file 3 **Evaluation of false positive rate of Haplo2Ped with completely simulated genotype data**. Comparison of false positive rate between Haplo2Ped and Merlin based on simulated genotype data of thirty different pedigree structures. Click here for file

Additional file 4 **Software comparison with simulated pedigree in incomplete penetrance**. The detected regions reported by Haplo2Ped and Merlin using simulated pedigree in incomplete penetrance. Click here for file
