# Supplementary material for: Haplo2Ped: a tool using haplotypes as markers for linkage analysis
Source: BMC Bioinformatics. 2011 Aug 22;12:350. doi: 10.1186/1471-2105-12-350 (PMC3179971; doi:10.1186/1471-2105-12-350)
Supplement: Additional file 1 — The pedigree simulation process. The pedigree simulation process was carried out based on a real genotype data set of a RP (retinitis pigmentosa) family. The family members' affected status was reset and six disease co-segregating haplotypes were simulated in chromosomes 1, 5, 9, 13, 17, and 21. [file 1471-2105-12-350-S1.DOC]

**The pedigree simulation process**

Before generating simulated disease targets, we first reset the affected status of family members based on the dominant model (main text Figure 3). All the genotype data in the six regions (expected regions shown in main text Table 1) were then simulated under Mendelian rules.

For each of the six assumed regions (main text Table 1), one of the two haplotypes from the disease founder (individual 2 in Figure 3) was considered as the disease-causative haplotype that harbors the causal mutation. The other haplotype of the disease founder was considered as the healthy haplotype without the causal mutation. We then modified the genotypes of SNPs in the six assumed regions in the second and third generation family members.

Taking the second generation members (individuals 4, 5, 6, 7, 9, and 10) as an example, for each simulation target region, we first determined the haplotypes that were transmitted from the father (individual 1) to the six children. Then, the disease-causative haplotype coming from the disease founder (individual 2, their mother) was assigned to the three affected children (individuals 4, 7, and 10) as the other parental haplotype. The mother’s healthy haplotype was assigned to the three healthy children (individuals 5, 6, and 9). The simulation process in the 3rd generation was similar to that in the 2nd generation.

To further test the detection power of Haplo2Ped, we designed crossovers at the middle of the simulation regions in chromosomes 17 and 21 in individuals 12 (affected member) and 15 (unaffected member), respectively. This was achieved by simulating a recombination event at the middle of the disease-causative haplotype in the mother (individual 4) of individual 12 and another recombination in the father (individual 7) of individual 15.
