# Supplementary material for: Haplo2Ped: a tool using haplotypes as markers for linkage analysis
Source: BMC Bioinformatics. 2011 Aug 22;12:350. doi: 10.1186/1471-2105-12-350 (PMC3179971; doi:10.1186/1471-2105-12-350)
Supplement: Additional file 2 — Software comparisons using real data. Linkage regions detected by three different softwares are from data of a real study (Table S1). The pedigree analyzed is a three-generation Han Chinese family with complex digital anomalies. [file 1471-2105-12-350-S2.DOC]

**Software comparison using real data**

The linkage regions detected by Haplo2Ped using data from a real study (Table S1). The pedigree analyzed is a three-generation Han Chinese family with complex digital anomalies. The three regions detected by Haplo2Ped with the maximum LOD scores across the whole genome are listed in the table (two false positive regions in chromosomes 12 and 16). The physical position of the disease-causative mutation is chr7: 41972312. It is a single nucleotide deletion (c.2884delG) in exon 14 of gene *GLI3*. This frame shift mutation generated a truncated protein with 40 non-endogenous amino acids at its C-terminal end. All the six regions reported by Merlin with LOD score greater than 1.70 are listed (five false positive regions). The disease-causative region (in chromosome 7) does not reach the maximum LOD score in Merlin. SNPLINK detected a region similar to the region detected by Haplo2Ped, but it did not give a LOD score.

**Table S1 Linkage regions reported by Haplo2Ped and other software in a study of real data.**

| **Chr** | **Haplo2Ped** | | **Merlin** | | **SNPLINK ^b^** |
| --- | --- | --- | --- | --- | --- |
|  | **Detected region**  **(bp)** | **LOD score** | **Detected region**  **(bp)** | **LOD score** |  |
| 7 ^a^ | 33,904,914-  45,529,271 | 2.107 | 34,513,811-  45,357,339 | 1.999 | 33,813,320-  45,489,972 |
| 12 | 8,863,236-  27,575,082 | 2.107 | 8,920,954-  27,384,004 | 1.931 | 8,912,180-  27,590,586 |
| 16 | 12,591,826-  47,595,720 | 2.107 | 13,022,862-  47,452,925 | 1.875 | 12,547,302-  47,563,323 |
| 4 | / | / | 92,592,351-  92,650,751 | 2.171 | / |
| 15 | / | / | 23,557,354-  23,570,131 | 2.008 | / |
| 20 | / | / | 40,836,396-  40,878,396 | 2.036 | / |

*^a^The disease-causative region.*

*^b^* *Regions detected by SNP4Linkage were the same as those found by SNPLINK.*
