# Supplementary material for: Haplo2Ped: a tool using haplotypes as markers for linkage analysis
Source: BMC Bioinformatics. 2011 Aug 22;12:350. doi: 10.1186/1471-2105-12-350 (PMC3179971; doi:10.1186/1471-2105-12-350)
Supplement: Additional file 3 — Evaluation of false positive rate of Haplo2Ped with completely simulated genotype data. Comparison of false positive rate between Haplo2Ped and Merlin based on simulated genotype data of thirty different pedigree structures. [file 1471-2105-12-350-S3.DOC]

**Evaluation of false positive rate of Haplo2Ped with completely simulated genotype data**

As shown in Figure S1, Merlin reports significantly more false positives than Haplo2Ped with 30 simulated independent pedigrees (*p-value* = 9.36E-07, t-test). The Perl script that was used to simulate the data set of a pedigree is available along with the Haplo2Ped package. In this program, genotypes of unrelated individuals were generated first by the random allocation of ATCG alleles. Genotypes of offspring were inferred according to Mendelian inheritance rules accompanied by specified numbers (~30 in male, ~40 in female) of random recombination events. One allele of a SNP was determined (disease allele) to be the causal mutation in one disease founder for each family. The haploid with the disease allele was inherited by the affected offspring, while the haploid with the healthy allele was transmitted to the unaffected ones.


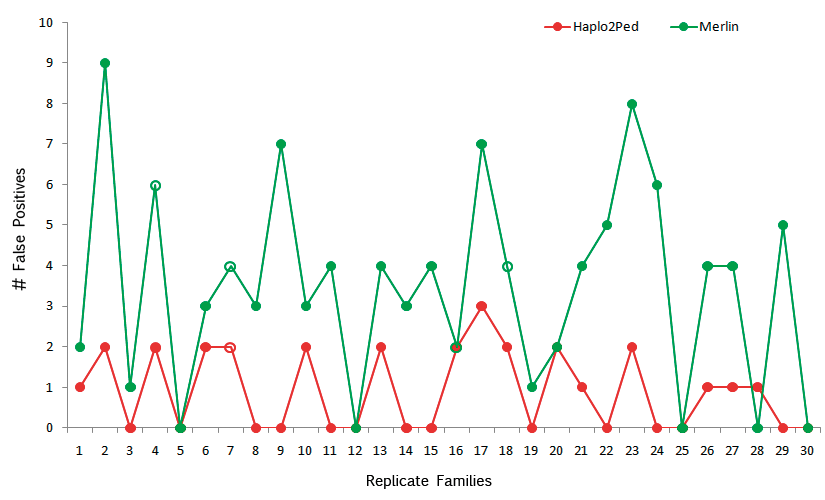


**Figure S1**. False positive regions detected by Haplo2Ped and Merlin using the simulation data. The x- and y-axes represent the false positive regions and the pedigree numbers respectively. The green line and circles denote the results by Merlin; the red line and circles denote the results by Haplo2Ped. Filled circles indicate that the software successfully located disease-causing region; open circles represent failure.
