# Supplementary material for: Haplo2Ped: a tool using haplotypes as markers for linkage analysis
Source: BMC Bioinformatics. 2011 Aug 22;12:350. doi: 10.1186/1471-2105-12-350 (PMC3179971; doi:10.1186/1471-2105-12-350)
Supplement: Additional file 4 — Software comparison with simulated pedigree in incomplete penetrance. The detected regions reported by Haplo2Ped and Merlin using simulated pedigree in incomplete penetrance. [file 1471-2105-12-350-S4.DOC]

**Software comparison with simulated pedigree in incomplete penetrance**

The detected regions reported by Haplo2Ped and Merlin using simulated pedigree with incomplete penetrance are shown in Table S2. We set the affected status of one affected family member (12) as unknown when performing the linkage analysis.

***Table S2*** *Linkage regions reported by Haplo2Ped and Merlin with the simulated pedigree in incomplete penetrance.*

| **Chr** | **Expected region^a^ (bp)** | **Haplo2Ped** | | **Merlin** | |
| --- | --- | --- | --- | --- | --- |
|  |  | **Detected region**  **(bp)** | **LOD score** | **Detected region (bp)** | **LOD score** |
| 1 | 216,655,820-  217,662,693 | 216,663,085-  217,668,715 | 3.010 | 217,005,036-  217,630,979 | 1.503 |
| 5 | 38,764,018-  41,787,459 | 38,631,518-  42,305,421 | 3.010 | 38,893,124-  41,665,719 | 1.503 |
| 9 | 27,316,060-  31,341,194 | 27,143,875-  31,602,602 | 3.010 | 27,320,515-  31,111,424 | 1.500 |
| 13 | 96,346,535-  101,352,382 | 96,328,971-  101,371,739 | 3.010 | 96,328,971-  101,304,534 | 1.500 |
| 17 | 50,661,600-  54,645,983 | 50,621,012-  54,774,779 | 3.010 | 50,690,828-  58,335,700 | 1.500 |
| 21 | 21,812,513-  26,812,202 | 21,818,487-  31,888,034 | 3.010 | 21,879,468-  31,771,430 | 1.500 |
| 1 | / | / | / | 55,549,288-  62,400,262 | 1.500 |
| 5 | / | / | / | 10,525,135-  11,769,406 | 1.500 |
| 9 | / | / | / | 73,494,327-  80,024,718 | 1.500 |

*^a^ Expected region is the simulated region assumed to contain the disease mutation.*
